# Evaluation of ultrasound-guided Freka-Trelumina enteral nutrition tube placement in the treatment of acute pancreatitis

**DOI:** 10.1186/s12876-020-1172-0

**Published:** 2020-01-29

**Authors:** Zhijun Liu, Jintao Guo, Weidong Ren, Shaoshan Tang, Ying Huang, Liping Huang, Siyu Sun, Lianjie Lin

**Affiliations:** 1 Ultrasound Department, Shengjing Hospital of China Medical University, Liaoning Province, People’s Republic of China; 2 Endoscopy Center Department, Shengjing Hospital of China Medical University, Liaoning Province, People’s Republic of China; 3 Gastroenterology Department, Shengjing Hospital of China Medical University, Liaoning Province, People’s Republic of China

**Keywords:** Acute pancreatitis, Enteral nutrition, Gastrointestinal decompression, Ultrasound

## Abstract

**Background:**

Enteral nutrition should be implemented as early as possible in patients with moderate or severe acute pancreatitis. This study was designed to evaluate the feasibility and Deffectiveness of ultrasound-guided Freka-Trelumina tube placement for enteral nutrition in acute pancreatitis.

**Methods:**

Patients with severe acute pancreatitis admitted to Shengjing Hospital of China Medical University who needed Freka-Trelumina tube placement for enteral nutrition and gastrointestinal decompression were included in the current study. The relevant evaluation indicators of tube placement included the success rate of tube placement, tube placement time, tube shift rate, and blocking rate. In addition, the evaluation indicators of ultrasound-guided tube placement (from 1 January 2018 to 31 July 2019) were compared with those of previous endoscope-guided placement (from 1 January 2015 to 31 December 2017) by analysing the data from the electronic medical record system.

**Results:**

The success rate of ultrasound-guided tube placement was 90.7% (49/54). All 49 patients tolerated the Freka-Trelumina feeding tube. The average ultrasound-guided tube placement time for the 49 patients was 18.4 ± 12.8 min (range, 5–36 min). The Freka-Trelumina feeding tube had a shift rate of 10.2% (5/49). The blocking rate of the Freka-Trelumina feeding tube was 12.2% (6/49). The success rate of tube placement, tube shift rate and blocking rate for endoscope-guided tube placement were 100% (62/62), 11.3% (7/62), and 12.9% (8/62), respectively. The average endoscope-guided tube placement time for the 62 patients was 16.5 ± 5.7 min (range, 12–31 min). The comparison between the ultrasound-guided group and the endoscope-guided group showed that the success rate of tube placement, tube placement time, tube shift rate and blocking rate were similar.

**Conclusion:**

The ultrasound-guided method can be done non-invasively at the bedside, which is safe and convenient, and the Freka-Trelumina feeding tube can be placed in time to achieve the goal of early enteral nutrition and gastrointestinal decompression.

## Background

Severe acute pancreatitis often occurs suddenly and progresses rapidly with numerous complications that can even lead to multiple-organ dysfunction, resulting in high mortality. Evidence-based medical evidence indicates that early enteral nutrition can preserve intestinal mucosal barrier function in patients with severe acute pancreatitis and reduce endotoxin translocation, pancreatic infection, the organ failure rate, and mortality [1–9]. Therefore, the guidelines for the diagnosis and treatment of acute pancreatitis recommend enteral nutrition as an important treatment, which should be implemented as early as possible in patients with moderate to severe acute pancreatitis [1–9].

There are currently three placement methods for Freka-Trelumina enteral nutrition tubes (blind, radiography-guided, and endoscope-guided placement) [10–15]. Compared with the traditional placement method, the bedside ultrasound-guided method can avoid the risks that come with patient transportation and the discomfort and damage associated with the gastroscope method, and it can be conveniently performed non-invasively at the bedside. This study was designed to evaluate the feasibility and effectiveness of ultrasound-guided Freka-Trelumina tube placement for enteral nutrition in acute pancreatitis.

## Methods

This study was approved by the institutional review board of Shengjing Hospital of China Medical University (2018PS027J), and informed consent was obtained from each patient or the next of kin. All experiments were carried out in accordance with the Declaration of Helsinki.

### Patients

Patients with severe acute pancreatitis admitted to Shengjing Hospital of China Medical University from 1 January 2018 to 31 July 2019 who needed Freka-Trelumina tube placement for enteral nutrition and gastrointestinal decompression were included in the current study. In addition, to compare the novel ultrasound-guided tube placement with previous endoscope-guided tube placement (from 1 January 2015 to 31 December 2017), we also analysed the data from the electronic medical record system.

### Material and equipment

The following materials and equipment were used in the current study: Freka-Trelumina feeding tube (Fresenius Kabi AG, Bad Homburg, Germany); vacuum suction chamber (length, 95 cm [end to the stomach]; inner diameter, CH16; outer diameter, 5.3 mm); pressure-regulating chamber (length, 95 cm [end to the stomach]); feeding chamber (length, 46 cm [end to the jejunum]; inner diameter, CH9; outer diameter, 2.9 mm; total length, 150 cm); ultrasound machine (Philips CX50; Amsterdam, Holland); high-frequency probe (L12–3 linear probe); abdominal probe (C5–1 convex probe); 1 dressing bowl; 1 package of gauze; 100 ml of warm water; 1 pair of sterile gloves; 1 piece of 50-ml syringe; 1 piece of sterile towel; and 1 piece of wide tape.

### Ultrasound-guided Freka-Trelumina enteral nutrition tube placement

**Fig. 1:**
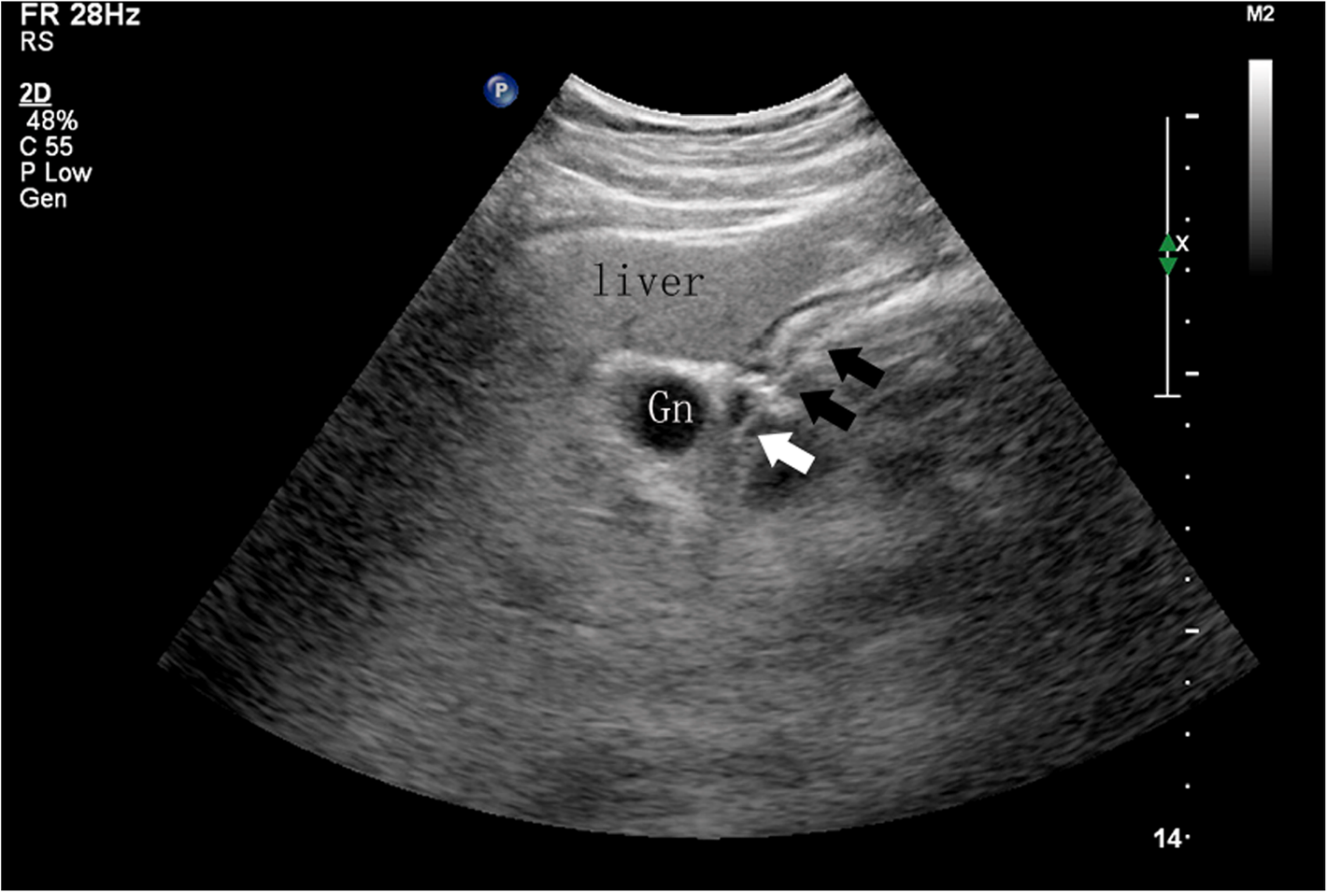
Freka-Trelumina tube with the guide wire (presenting as a linear hyperecho) entering the duodenum through the antral pylorus. When the catheter depth reaches 75 cm, ultrasound should demonstrate that the Freka-Trelumina tube with the guide wire (presenting as a linear hyperecho) has entered the duodenum (white arrow) through the antral pylorus (black arrows). Gn, gallbladder neck

**Fig. 2:**
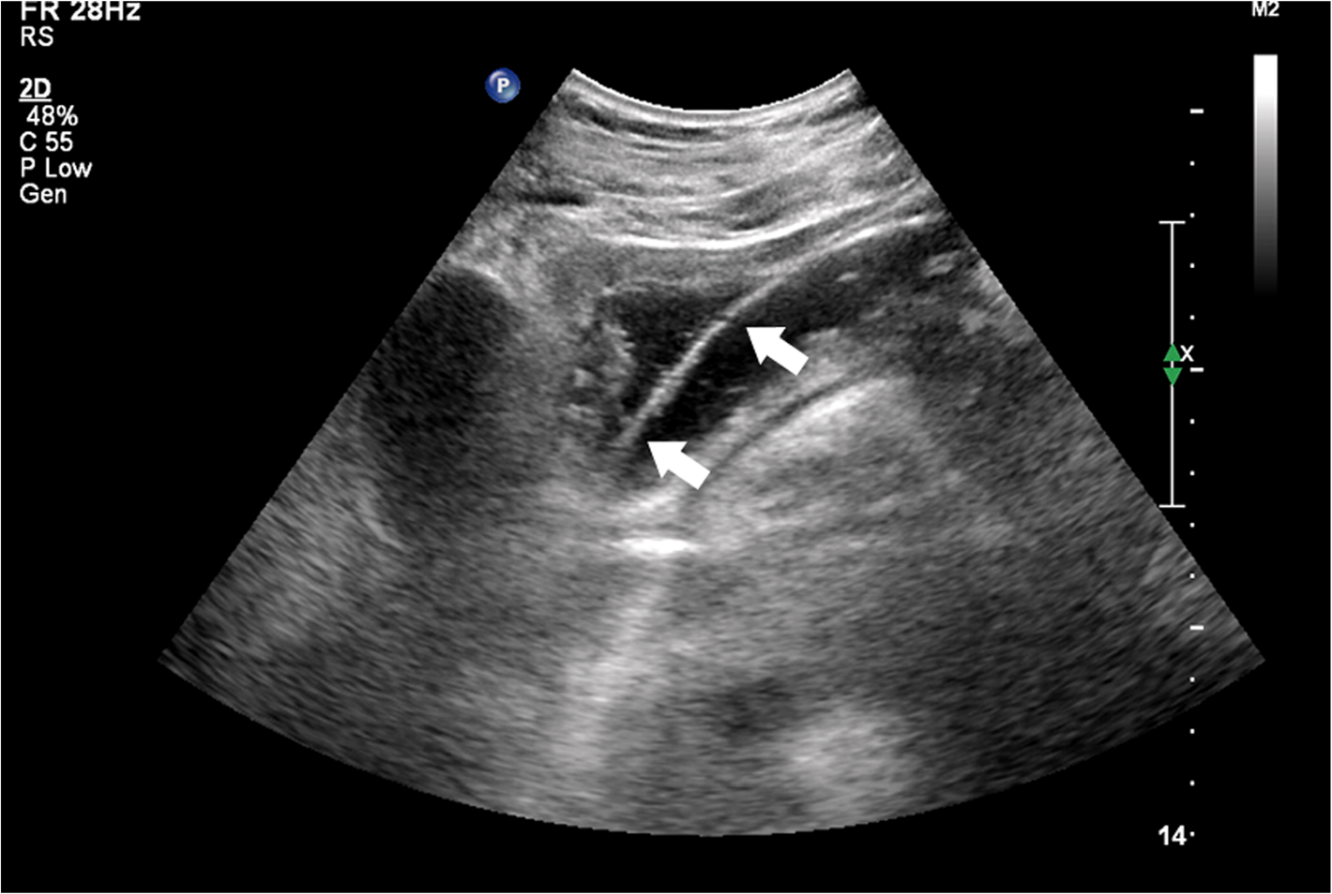
Freka-Trelumina tube with guide wire (presenting as a linear hyperecho) within the antrum. If the quality of the ultrasound image is poor, 100 ml of saline should be injected into the gastric lumen of the Freka-Trelumina tube to improve the image quality. The ultrasound view shows that the Freka-Trelumina tube with guide wire presents as a linear hyperecho (arrow) within the antrum

**Fig. 3:**
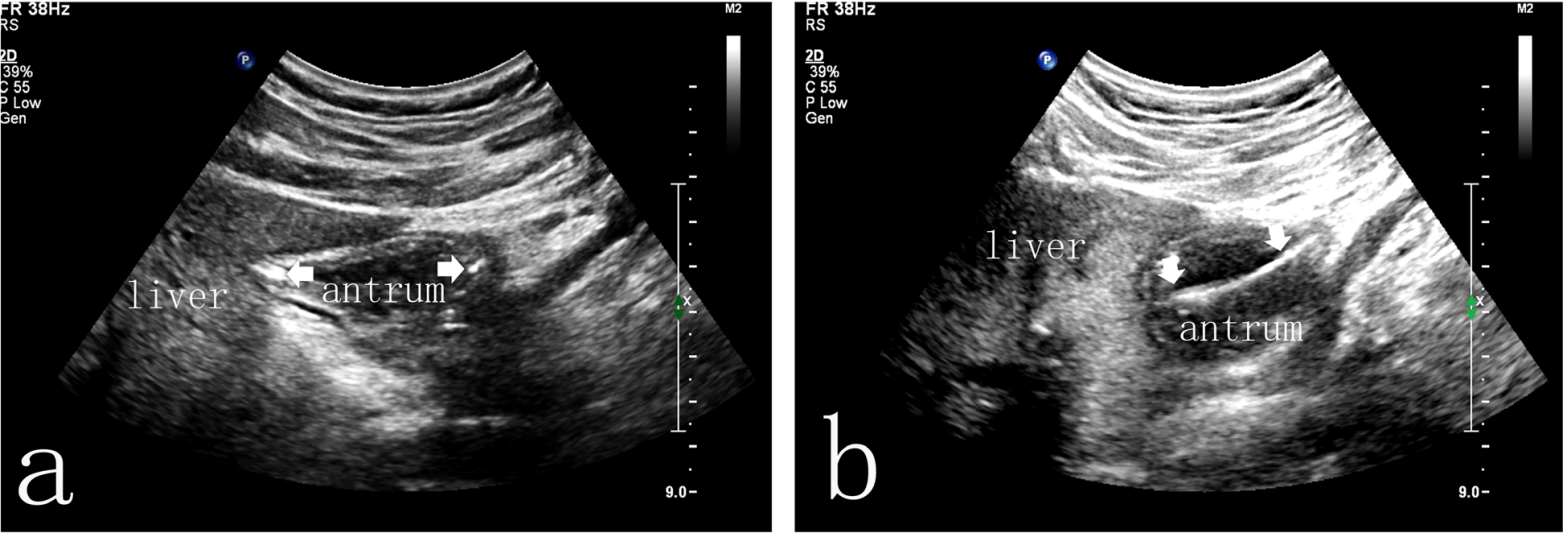
Freka-Trelumina tube curving in the stomach. **a** The ultrasound view shows that the Freka-Trelumina tube presents as two hyperechoic points against the wall of the stomach, indicating that the tube curves in the stomach. **b** Continuing to scan along the gastric cavity shows that the Freka-Trelumina tube presents as a linear hyperecho against the wall of the stomach (indicating that the tube is reflexed here)

**Fig. 4:**
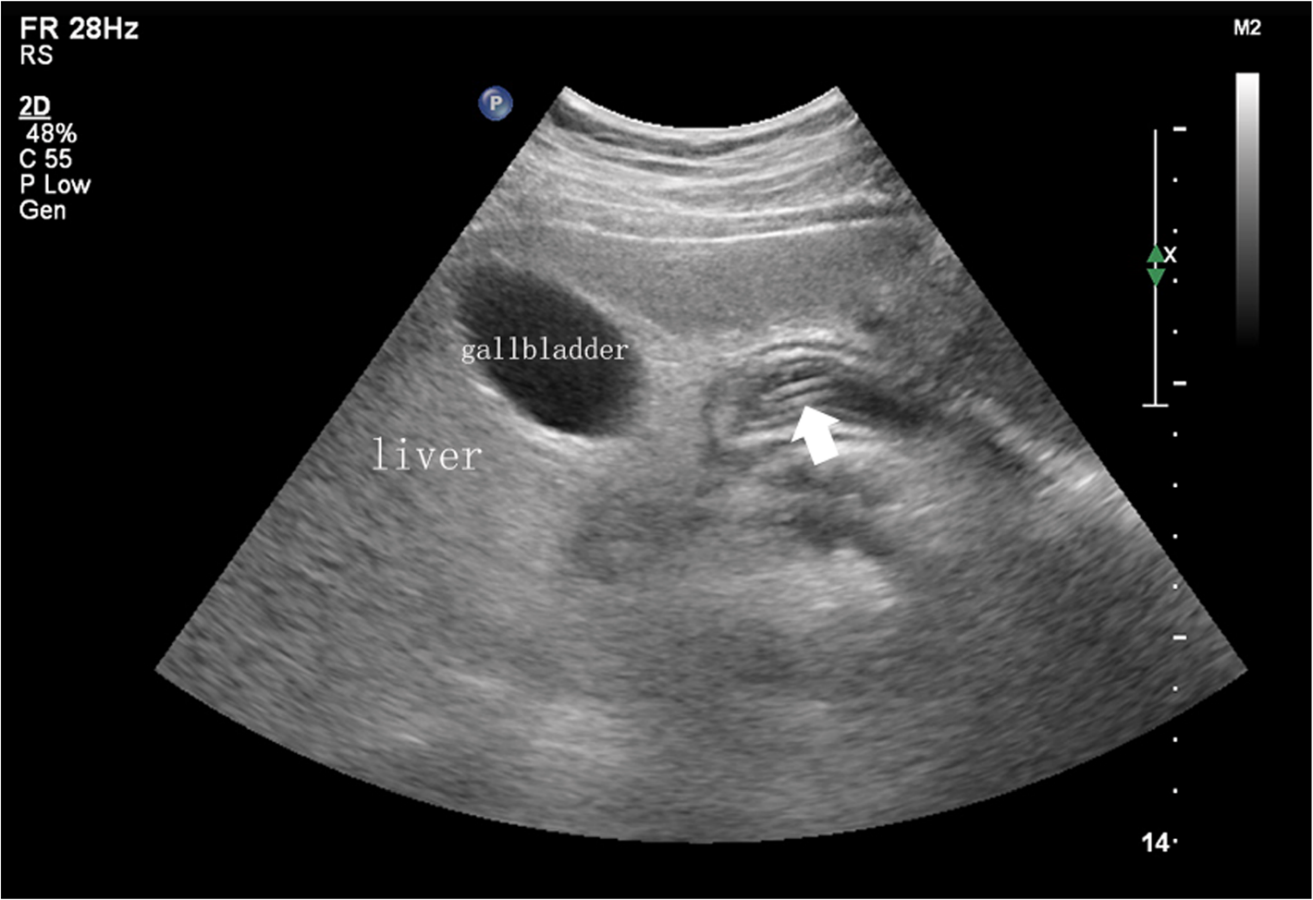
Ultrasound examination shows a “parallel tubular echo” (arrow) after the guide wire is withdrawn

A sterile towel was spread on the operating room table, and warm water was poured into the dressing bowl. After donning sterile gloves, paraffin was used to lubricate the surface of the Freka-Trelumina feeding tube. A guide wire was inserted into the Freka-Trelumina feeding tube, which not only maintained the tension of the feeding tube but also facilitated the spiral advancement of the tube. Furthermore, the guide wire was more clearly displayed under ultrasound (the guide wire appeared as a linear hyperecho). The placement of the Freka-Trelumina feeding tube was divided into two major steps. The first step was to place the Freka-Trelumina tube into the stomach, which was similar to gastric tube placement. The second step was to introduce the Freka-Trelumina tube along the stomach greater curvature into the duodenum through the pylorus under ultrasound guidance, mainly by rotation and propulsion. The operating points for the first step of placing the Freka-Trelumina feeding tube into the stomach were as follows: the patient’s nostrils were cleaned, and the nasal passage with good ventilation was selected; gauze was placed on the left hand to hold the tube, and the tip of the tube was held with tweezers in the right hand; the tube was inserted along the naris and advanced slowly until the tube was in the throat (a depth of 14–16 cm); the tube placement was continually promoted to a depth of 55 cm; and if the patient was conscious, the patient was asked to swallow repeatedly. Whether the Freka-Trelumina tube was curled in the mouth was determined, and whether the tube was in the stomach was checked ultrasonically. Ultrasound should show the Freka-Trelumina tube as a linear hyperecho in the stomach. The operating points for the second step of advancing the Freka-Trelumina tube into the duodenum through the pylorus included the following: the sonographer placed the abdominal probe near the neck of the gallbladder to observe the antral pylorus; the assistant continued to advance the Freka-Trelumina feeding tube; when the catheter depth reached 75 cm, ultrasound should demonstrate that the Freka-Trelumina tube with the guide wire (presenting as a linear hyperecho) entered the duodenum through the antral pylorus (Fig. 1); if the quality of the ultrasound image was poor, 100 ml of saline was injected into the gastric lumen of the Freka-Trelumina tube to improve the image quality (Fig. 2); if the ultrasound did not show the Freka-Trelumina tube passing through the pylorus when the depth of the catheter was 75 cm, it may have been curved in the stomach, and the ultrasound could show the Freka-Trelumina tube curving in the stomach (Fig. 3); at this point, the Freka-Trelumina tube was withdrawn to a depth of 55 cm, then rotated and propelled again; and after the Freka-Trelumina tube passed through the pylorus smoothly, the assistant continued to advance the catheter until the catheter depth reached 115 cm. Then the guide wire was slowly withdrawn. Ultrasound examination showed a “parallel tubular echo” image after the guide wire was withdrawn (Fig. 4). Finally, the Freka-Trelumina feeding tube was fixed to the cheek of the patient with tape. Bedside abdominal X-ray was used as the gold standard to determine successful placement of the Freka-Trelumina tube in the upper part of the jejunum.

### Evaluation indicator

The relevant evaluation indicators of tube placement included the success rate of tube placement, the number of tube advances before passing through the pylorus, tube placement time, tube shift rate, and blocking rate. Tube placement was considered successful if ultrasound showed that the tube successfully passed through the pylorus or the end of the tube was located in the jejunum. If the tube placement time exceeded 60 min and the tube was not confirmed to have passed through the pylorus, the tube placement was considered a failure. If the tube is curved in the stomach when the tube is advanced, the tube should be slightly retracted and then re-advanced to pass the pylorus, and the number of advances was recorded once per adjustment. The time when the Freka-Trelumina tube entered the nasal cavity was the initiation time of tube placement, and the completion time was when ultrasound observation confirmed that the tube had passed through the pylorus. After the tube had been successfully placed, the depth of the tube was checked every 6 h after marking the depth of the tube end at the patient’s nose. If the tube shift upside was < 10 cm, the tube was adjusted manually, and an imaging examination (ultrasound or abdominal X-ray) was used to confirm that the tube had been readjusted to an adequate position (no shift). The tube was considered to be seriously shifted with the shift distance was > 10 cm, and it needed to be re-inserted and counted into the shift rate. If there was obvious obstruction at the stomach or jejunum end that could not be dredged after the introduction of gas or liquid and/or guide wire dredging, the tube was considered blocked.

Patient tolerance at the time of tube placement and after tube placement was as follows: grade I, no special discomfort; grade II, mild discomfort, but tolerable; grade III, severe discomfort, barely tolerable; and grade IV, severe discomfort, intolerable. The white blood cell count, serum amylase, and C-reactive protein were routinely monitored. The clinical symptoms of patients were observed during the treatment, and complications of the eternal nutrition tube were also observed (haemorrhage, arrhythmia, aspiration, and regurgitation).

## Results

A total of 54 patients were enrolled, including 38 males and 16 females (age range, 22–86 years; mean age, 44 ± 14.8 years). The body mass index range was 23.9–36.9 kg/m^2^, with a mean BMI of 27.6 kg/m^2^. There were 30 cases of biliary pancreatitis and 19 cases of hyperlipidaemic pancreatitis. The aetiology of 5 cases was not completely clear.

### Evaluation results of tube placement-related indicators

The success rate of ultrasound-guided tube placement was 90.7% (49/54). Among the 49 patients who underwent successful ultrasound-guided tube placement, the Freka-Trelumina tube passed the pylorus with 1 advancement in 13 patients (26.5% [13/49]). In another 17 cases (34.7% [17/49]), the tube needed to be slightly retracted and passed the pylorus on the second attempt. The other 19 cases (38.8% [19/49]) needed more than two attempted advancements to pass the pylorus. Ultrasound directly revealed the Freka-Trelumina tube within the duodenum or jejunum in 20 cases (40.8% [20/49]). Ultrasound did not directly demonstrate tube placement within the duodenum or jejunum in another 29 cases (59.2% [29/49]) because of intestinal distension, abdominal fat thickness, and other factors. Ultrasound-guided tube placement failed in 5 cases, for a failure rate of 9.3% (5/54), and the tube was ultimately placed with gastroscope assistance. The average ultrasound-guided tube placement time for the 49 patients was 18.4 ± 12.8 min (range, 5–36 min). The Freka-Trelumina feeding tube had a shift rate of 10.2% (5/49). The blocking rate of the Freka-Trelumina feeding tube was 12.2% (6/49).

All 49 patients tolerated the Freka-Trelumina feeding tube. Seventeen, 23, 9, and zero patients reported grade I, II, III, and IV tolerance, respectively. After treatment, the white blood cell count, serum amylase, and C-reactive protein levels gradually decreased. Abdominal pain and bloating symptoms were relieved slowly. The complications of ultrasound-guided tube placement were as follows: haemorrhage (0/54), arrhythmia (0/54), aspiration (0/54), and regurgitation (0/54).

In the case of previous endoscope-guided tube placement, the success rate of tube placement, tube shift rate and blocking rate were 100% (62/62), 11.3% (7/62), and 12.9% (8/62), respectively. The average endoscope-guided tube placement time for the 62 patients was 16.5 ± 5.7 min (range, 12–31 min). The complications of endoscope-guided tube placement were as follows: haemorrhage (2/62), arrhythmia (4/62), aspiration (1/62), and regurgitation (2/62).

As shown in Table 1, the comparison between the ultrasound-guided group and the endoscope-guided group showed that the tube placement time (18.4 ± 12.8 vs 16.5 ± 5.7, *p* > 0.05), tube shift rate (10.2% vs 11.3%, *p* > 0.05) and blocking rate (12.2% vs 12.9%, *p* > 0.05) were similar; although the success rate in the ultrasound-guided group was slightly lower (90.7% VS 100%, *P* < 0.05), the results were also similar.

**Table 1:** The comparison between the ultrasound-guided group and the endoscope-guided group

Group	Success rate(%)	Placement time(min)	Shift rate(%)	Blocking rate(%)
Ultrasound-guided	90.7% (49/54)	18.4 ± 12.8	10.2% (5/49)	12.2% (6/49)
Endoscope-guided	100% (62/62)	16.5 ± 5.7	11.3% (7/62)	12.9% (8/62)

## Discussion

The guidelines for the diagnosis and treatment of acute pancreatitis suggest that enteral nutrition should be an important part of treatment and should be implemented as early as possible in patients with moderate or severe acute pancreatitis. Although gastric feeding is normally sufficient and safe in most patients with acute pancreatitis, nasogastrojejunal tube can reduce the probability of reflux and aspiration, increase the nutrient utilization ratio, and provide adequate nutrition within a short time [16, 17]. Use of jejunal tubes can improve nutrition and reduce gastric reflux and thereby probably prevents aspiration of nutrition fluid [16, 17]. A spiral nasal jejunal nutrition tube is the most commonly used enteral nutrition method [15–17], but it is still difficult to avoid the problem of reflux aspiration of gastric fluid. Therefore, double tube placement with continuous gastric decompression and nasal jejunal tubes is a traditional and common enteral nutrition pathway for patients with severe acute pancreatitis. Most patients find this “two-pronged” model difficult to tolerate. The Freka-Trelumina feeding tube can meet the requirements of simultaneous gastrointestinal decompression and enteral nutrition. The Freka-Trelumina feeding tube not only solves the gastrointestinal decompression needed in patients with pancreatitis but also resolves pancreatic secretion problems by jejunal feeding because consuming food in the head, stomach, and duodenum might increase pancreatic secretion [15].

Currently, there are three methods for placement of a Freka-Trelumina feeding tube (blind, DSA-guided, and gastroscope-guided placement). The success rate of blind placement is only 20%. The disadvantages of radiography-guided placement are as follows: 1) risks that come from transportation, because some critically ill patients need ventilator maintenance; and 2) waiting time for the radiography room limits the use of this method [16, 17]. Gastroscope-guided tube placement can be performed at the bedside, which is a commonly used method of tube placement, but it also has the following disadvantages: 1) it is invasive; 2) the tube can only be placed into the duodenum under gastroscopy, and the gastroscope view does not extend beyond the duodenojejunal junction; and 3) after successful placement under gastroscopy, the friction between the gastroscope and the three-lumen tube may bring the Freka-Trelumina feeding tube out of position, thus increasing the operation number and the difficulty of gastroscope-guided tube placement [15–17].

With improvements in ultrasound techniques, transabdominal ultrasound has been used in the initial screening of gastric diseases for patients unwilling to undergo gastroscopy [18–24]. In the fasting state, the pylorus of most patients can be detected beneath the gallbladder neck by transabdominal ultrasound, which makes it possible to insert a feeding tube through the pylorus into the duodenum under the guidance of transabdominal ultrasound.

This study showed that the success rate of ultrasound-guided Freka-Trelumina feeding tube placement was as high as 90.7% (49/54). In this study, ultrasound-guided tube placement failed in 5 patients. The causes of failure included excessive gastroptosis or excessive angulation of the gastric cavity, which resulted in repeated reflexion of the Freka-Trelumina tube in the gastric cavity, thus increasing the difficulty in inserting the tube through the pylorus. For cases with failed placement, traditional gastroscope-guided tube placement can be performed without increasing any risk to the patient.

Because reflexion of the tube within the duodenum is rare, when the ultrasound accurately shows that the Freka-Trelumina feeding tube passes through the pylorus, the tube can be successfully placed into the jejunum by continuous advancement of the tube. Although ultrasound directly displayed the Freka-Trelumina feeding tube within the jejunum in 20 cases (40.8% [20/49]) in this study, it displayed the tube successfully passing through the pylorus in all cases. A lower display rate of the jejunum by ultrasound does not influence the application of the ultrasound-guided technique.

This study demonstrated that the ultrasound-guided Freka-Trelumina feeding tube placement method is safe and convenient. Compared with radiography-guided and endoscope-guided tube placement, the Freka-Trelumina feeding tube method avoids the risk of radiation damage and transportation as well as the discomfort of gastroscope-guided placement. The Freka-Trelumina feeding tube method does not rely on the endoscopist or have to wait for the radiography room. Using this method, the tube can be placed in time to achieve the goal of early enteral nutrition and gastrointestinal depression, which can reduce the infection rate and hospital stay. In terms of operative duration, the average tube placement time of 49 patients in this study was 18.4 ± 12.8 min. By comparison, the average previous endoscope-guided tube placement time for the 62 patients was 16.5 ± 5.7 min.

The tube shift rate and blocking rate for the novel ultrasound-guided tube placement were 10.2% (5/49) and 12.2% (6/49), respectively. By comparison, the tube shift rate and blocking rate for the endoscope-guided tube placement were 11.3% (7/62) and 12.9% (8/62), respectively.

The comparison between the ultrasound-guided group and the endoscope-guided group showed that the success rate of tube placement, tube placement time, tube shift rate and blocking rate were similar.

## Conclusions

In summary, the ultrasound-guided method can be done non-invasively at the bedside, which is safe and convenient, and the Freka-Trelumina feeding tube can be placed in time to achieve the goal of early enteral nutrition and gastrointestinal decompression. Early enteral nutrition can reduce the infection rate and hospital stay, which have a positive role in the treatment of severe acute pancreatitis. This result should be evaluated further by means of randomized controlled trials and economic evaluation because the small sample size and retrospective study design are obvious limitations.

## Data Availability

The datasets used and analysed during the current study are available from the corresponding author on reasonable request.
